# Characteristics of biological control and mechanisms of *Pseudomonas chlororaphis* zm-1 against peanut stem rot

**DOI:** 10.1186/s12866-021-02420-x

**Published:** 2022-01-05

**Authors:** Fengying Liu, Shan Yang, Fenghua Xu, Zhen Zhang, Yifang Lu, Juanmei Zhang, Gang Wang

**Affiliations:** 1 Institute of Microbial Engineering, Laboratory of Bioresource and Applied Microbiology, School of Life Sciences, Henan University, Kaifeng, 475004 China; 2 Engineering Research Center for Applied Microbiology of Henan Province, Kaifeng, 475004 China; 3 School of Pharmaceutical, Henan Univeristy, Kaifeng, 475004 China; 4 School of Life Sciences, Henan University, Jinming Street, Kaifeng, 475004 Henan People’s Republic of China

**Keywords:** *Pseudomonas chlororaphis*, Peanut stem rot, *Sclerotium rolfsii*, Phenazine, Biocontrol efficacy

## Abstract

**Background:**

Peanut stem rot is a serious plant disease that causes great economic losses. At present, there are no effective measures to prevent or control the occurrence of this plant disease. Biological control is one of the most promising plant disease control measures. In this study, *Pseudomonas chlororaphis* subsp. *aurantiaca* strain zm-1, a bacterial strain with potential biocontrol properties isolated by our team from the rhizosphere soil of *Anemarrhena asphodeloides*, was studied to control this plant disease.

**Methods:**

We prepared extracts of *Pseudomonas chloroaphis* zm-1 extracellular antibacterial compounds (PECEs), determined their antifungal activities by confrontation assay, and identified their components by UPLC-MS/MS. The gene knockout strains were constructed by homologous recombination, and the biocontrol efficacy of *P. chlororaphis* zm-1 and its mutant strains were evaluated by pot experiments under greenhouse conditions and plot experiments, respectively.

**Results:**

*P. chlororaphis* zm-1 could produce extracellular antifungal substances and inhibit the growth of *Sclerotium rolfsii*, the main pathogenic fungus causing peanut stem rot. The components of PECEs identified by UPLC-MS/MS showed that three kinds of phenazine compounds, i.e., 1-hydroxyphenazine, phenazine-1-carboxylic acid (PCA), and the core phenazine, were the principal components. In particular, 1-hydroxyphenazine produced by *P. chlororaphis* zm-1 showed antifungal activities against *S. rolfsii*, but 2-hydroxyphenazine did not. This is quite different with the previously reported. The extracellular compounds of two mutant strains, Δ*phzH* and Δ*phzE*, was analysed and showed that Δ*phzE* did not produce any phenazine compounds, and Δ*phzH* no longer produced 1-hydroxyphenazine but could still produce PCA and phenazine. Furthermore, the antagonistic ability of Δ*phzH* declined, and that of Δ*phzE* was almost completely abolished. According to the results of pot experiments under greenhouse conditions, the biocontrol efficacy of Δ*phzH* dramatically declined to 47.21% compared with that of wild-type *P. chlororaphis* zm-1 (75.63%). Moreover, Δ*phzE* almost completely lost its ability to inhibit *S. rolfsii* (its biocontrol efficacy was reduced to 6.19%). The results of the larger plot experiments were also consistent with these results.

**Conclusions:**

*P. chlororaphis* zm-1 has the potential to prevent and control peanut stem rot disease. Phenazines produced and secreted by *P. chlororaphis* zm-1 play a key role in the control of peanut stem rot caused by *S. rolfsii*. These findings provide a new idea for the effective prevention and treatment of peanut stem rot.

**Supplementary Information:**

The online version contains supplementary material available at 10.1186/s12866-021-02420-x.

## Introduction

Peanut is one of the world’s most important oil crop species, as its cultivation area is second only to that of rapeseed, and plays an important role in oil production worldwide. Peanut stem rot is an important soil-borne fungal disease occurring in plants that leads to yield losses of many important crop species, including cultivated peanut [2, 15]. This fungal disease has been reported in many peanut-producing regions of the world, such as India, the United States, Argentina, Indonesia, the Philippines, Thailand, Vietnam and South Africa [18]. In China, peanut stem rot disease occurred widely in peanut-producing areas of Anhui Province from 1957 to 1959, and the incidence area surpassed 70% in three years [30]. In contrast, in recent decades, the occurrence of peanut stem rot disease has become exacerbated; the distribution area of the disease has been expanding annually; and the disease has been widespread in some producing areas of Shandong, Liaoning, Henan, Guangdong, Jiangxi and other provinces in China [1].

*Sclerotium rolfsii*, a pathogenic fungus, is the main pathogen causing peanut stem rot disease [13]. Unfortunately, *S. rolfsii* is very difficult to control, as it produces sclerotia that overwinter in the soil and cause disease in the following season [13, 19]. On the other hand, *S. rolfsii* can cause stem rot in more than 600 plants, especially cash crops such as peanut, onion, cotton, potato, soybean, tomato, wheat, and cucurbits [4, 11]; Therefore, it is a pathogenic fungus with diverse hosts [29]. Once peanut plants are infected with *S. rolfsii*, branch wilting and even whole-plant wilting can occur. Peanut stem rot caused by *S. rolfsii* is also known as southern stem rot, southern blight, white mould, and Sclerotium rot [29], which causes severe economic losses to current agricultural production and development.

At present, chemical fungicides were often used to control peanut stem rot, and agronomic measures, such as rotation with nonhost crops or coverage of infected crop debris via deep ploughing were also adopt [7]. Although these strategies can be employed to control this disease, it was still easy to get rid of control, and then spread. This is because chemical fungicides are not effective in the late diagnosis of the disease. Horticultural measures can only focus on prevention and cannot deal with the situation of diseased plants, too. For another, *S. rolfsii* has wide range of hosts, profuse mycelia, abundant persistent sclerotia, and genetic variability, once an infection occurs, it is difficult to eliminate the damage of this disease [25]. Furthermore, applying chemical fungicides causes many serious problems, such as fungicide residue, pathogen resistance and environmental pollution [13]. Hence, we urgently need an effective and eco-friendly control method to prevent the spread of *S. rolfsii*. The search for biological control of plant pests and diseases holds great promise as a safer and more environmentally friendly alternative to the use of chemical pesticides and others.

*Pseudomonas* has been reported to have good inhibitory activity against plant pathogens and has proved to be an important candidate of biocontrol strains [28]. Most plant-beneficial Pseudomonad strains were selected for their ability to suppress plant diseases. It was found that these Pseudomonad strains can produce a variety of secondary metabolites with antifungal activity [10]. The antifungal compounds produced by these biocontrol agents included the secretion of phenazine-1-carboxylic acid (PCA), 2,4-diacetylphloroglucinol, pyrrolnitrin, hydrogen cyanide, siderophores, and hydrolytic enzymes such as chitinases, proteases, cellulase, and β-glucanases [3]. According to previous reports, phenazine and its derivatives, a nitrogen-containing heterocyclic redox agents with broad-spectrum activity against gram-positive and gram-negative bacteria and fungi, are produced mainly by *Pseudomonads* and *Streptomyces* species [3, 12]. However, there are still few reports on biocontrol strains with excellent performance that can be used for the control of peanut stem rot, and the screening of efficient biocontrol strains is still the focus of current research. In this study, to find an effective way to control peanut stem rot plant disease, we aimed to screen biocontrol strains with activities against *S. rolfsii*. *P. chlororaphis* has also been reported to be a biocontrol strain of bacteria due to its production of various antibacterial substances, but its biocontrol characteristics and mechanisms are still unclear. In this paper, we further discussed the basic biocontrol of *P. chlororaphis* zm-1 and its ability to promote the control of peanut stem rot.

## Materials and methods

### Materials, test strains, and culture media

*P. chlororaphis* zm-1 was isolated from the rhizosphere soil of healthy plants and identified via determining its 16S rRNA sequence and sequence alignment analysis. The derived strains were constructed by homologous recombination. *P. chlororaphis* zm-1 and its derived strains were cultured in Luria-Bertani (LB) media, which included 10 g of peptone, 5 g of yeast extract, and 10 g of NaCl per litre. *S. rolfsii* was cultured on potato dextrose agar (PDA). The peanut Yuhua 12 used in this experiment is a cultivated variety, which was identified to belong to the Angiospermae, Leguminosae, Arachis, and its scientific name is *Arachis hypogaea* Linn. Its seeds are from the Plant Germplasm Resources and Genetic Engineering Center of Henan University, and it has been cultivated for 10 years. The peanut plants are grown in a greenhouse using pot culture, and plot-based experiments were carried out by simulating field planting.

### Confrontation assay under laboratory conditions

The antifungal activity of *P. chlororaphis* zm-1 against *S. rolfsii* (24 h old) was determined by a dual confrontation assay as described by Chen [2]. For details, *S. rolfsii* was inoculated on PDA plates and cultured at 25°C until the mycelia expanded and covered the whole plate. Agar blocks with a diameter of 6 mm were taken from the activated *S. rolfsii* culture with a sterilized hole punch and then inoculated onto the centre of a new PDA plate. Logarithmic-stage *P. chlororaphis* zm-1 (2 μL) was plated at a distance of 2 cm from the centre of the *S. rolfsii* disc. The suspension of *P. chlororaphis* zm-1 was inoculated at a distance of 2 cm from the centre of the plate at a volume of 10 μL, and a plate with the same amount of methanol was used as the control. The plates were incubated at 25 ± 2°C for 3 days and monitored regularly after 24 h of incubation for the zone of inhibition. The fungal inhibition rate is equal to the mycelial extension length after inhibition compared to that without inhibition. The differences in fungal inhibition rate were analysed by one-way analysis of variance (ANOVA) followed by Tukey’s pairwise post-hoc comparisons.

### Preparation of *P. chlororaphis* zm-1 extracellular compounds extract (PECE)

The extracellular antibacterial compounds of *P. chlororaphis* zm-1 were extracted from the culture media of the tested strains by the method of Peng et al. [22]. Briefly, cultures were grown for two days in 100 ml of potato dextrose broth (PDB) at 25°C. After cultivation for 3 days, the fermented broth was extracted with an equal volume of ethyl acetate three times. Finally, 10 g of crude extract was harvested after the organic phase was evaporated under vacuum pressure at 37°C. The concentrated crude metabolites were dissolved in methanol, the major compounds were purified by preparative scale chromatography equipped (Waters, USA) with a Reversed-phase C_18_ column (5.0 × 250 mm, with 2.5 μm coating layer), and their antibacterial activity was evaluated by confrontation assays.

### Components analysed and prepared by HPLC

The components separated and prepared by HPLC (Shimadzu, Japan) refer to the method of previous report [33]. 1mL of sample was loaded, and the temperature of the column box was set to 30 °C. The mobile phase was A: HPLC grade H_2_O (0.1% formic acid); B: HPLC grade acetonitrile. The chromatographic separation program was: 10% acetonitrile elution for 2 min, then was increased to 80% within 10 min, and finally increased to 100% within 3 min. The flow rate was 0.3 mL/min.

### Components identified by UPLC-MS/MS

According to our previous report [34], composition analyses were carried out by UHPLC-MS/MS using a Q-Exactive Plus mass spectrometer (Thermo Fisher, Waltham, USA) coupled to a Vanquish Flex system equipped with a Hypersil GOLD column (2.1 × 100 mm, with a 1.9 μm coating layer). 1mL of sample was loaded, and the temperature of the column box was set to 25°C. The A and B mobile phases consisted of H_2_O (including 0.1% formic acid) and acetonitrile, respectively. The chromatographic separation programme was as follows: 10% acetonitrile elution for 2 min, an increase to 80% within 10 min, and, finally, an increase to 100% within 3 min. The flow rate was 0.3 mL/min.

The mass spectrometric conditions were described as previously reports [34]. For detail, The mass spectrometer was operated in full scan mode and in both positive and negative modes in a range of *m/z* 50–1050. The resolution of the MS data was set to 70,000; the spray voltage was set to 3.2 kV for negative mode; the capillary temperature was set to 320°C; the auxiliary gas heater temperature was 350°C; the sheath gas flow rate was 35 L/h; the auxiliary gas flow was set to 15 L/h; the S-lens RF level was 50; the maximum injection time used was 100 ms; and the resolution of MS/MS acquisition was set to 17,500. The top eight ions in each full scan were isolated within a 1.0 Da window and then fragmented with stepwise collision energies of 20, 40 and 60 units and a maximum injection time of 50 ms, with an automatic generation control target of 10^6^. The acquired raw data were analysed using Compound Discover 3.2 (Thermo Fisher, Waltham, USA) in conjunction with information from several metabolite databases (mzCloud, mzVault, MassList, and ChemSpider).

### Construction of the gene deletion strains Δ*phzH* and Δ*phzE*

Whole-genome sequencing results (GenBank ID: CP048051) and subsequent gene functional annotations showed that the *P. chlororaphis* zm-1 genome encodes two proteins, PhzH (Protein ID: WP_038575848.1) and PhzE (Protein ID: WP_081359794.1), which are annotated as a phenazine biosynthesis-related protein and a phenazine-specific anthranilate synthase, respectively. We constructed gene knockout mutants of ∆*phzH* and Δ*phzE* by allelic exchange according to a previous report, with some modifications [32]. In detail, two fragments suitable for allelic exchange were created by cloning two *Eco*RI-*Hind*III DNA fragments containing locus-specific flanking regions into the *Eco*RI site of pK18 plasmid. These fragments were created via PCR using the primers listed in Table S1. Ligation of the two fragments led to the precise deletion of the respective open reading frame from the start to the stop codons and to generation of a *Hind*III site at the locus. Then, the pK18 plasmid was cloned into the *Escherichia coli* 116 competent cells. The plasmids were digested by *Eco*RI-*Hind*III enzymes and then amplified by PCR to verify the correctness of the cloned DNA fragment. The plasmid that contained the correct DNA fragment was demethylated and cloned into *E. coli* S17-1 by electroporation. The correct DNA fragment was purified again and then transformed into competent *P. chlororaphis* zm-1 cells. After recovery, the electroporation-competent cells were spread onto LB agar plates with 20 μg/mL kanamycin. After incubation at 30°C overnight, the correct clones were obtained and used for allelic exchange. Then, the correct mutant strains were screened on plates for two types of resistance.

### Biological control assays under greenhouse conditions

To evaluate the efficiency of *P. chlororaphis* zm-1 in controlling peanut stem rot, pot experiments were carried out under greenhouse conditions according to the previous reports by Chen et al. [2] and Dai et al. [5] with some modifications. Specifically, wheat grain sand medium with *S. rolfsii* were prepared as follows: Wheat grains were put into a triangular flask and soaked in distilled water for 3 h. The water was removed, and a 1/6 volume of sand was added. The mixture was autoclaved and then inoculated with *S. rolfsii*. Afterwards, the mixture was cultured at 30°C for one week until mycelia grew on all the wheat grains and then stored at 4°C. The peanut seeds were transferred to petri dishes for germination for 12 h and subsequently sown in pots containing 150 g of sterilized sandy soil. After growing for 14 days at 28±1°C, peanut seedlings with consistent growth were chosen for pot experiments. Then, the sand with the wheat grains and with *S. rolfsii* was ground with an aseptic mortar and sprinkled evenly onto the surface of the culture pots of 14 d old peanut seedlings, at a rate 10 g per pot. A 2 mL aliquot of the *P. chlororaphis* zm-1 suspension, suspended in sterile water and diluted to 1 ×10^8^ CFU/mL, was prepared and evenly sprayed onto the peanut shoots. Sterile water was used as a blank control, and carbendazim was used as a fungicide control. Each treatment involved 10 pots and was performed in triplicate. All the pots were kept at 28°C with the day 14 h and night 10 h in a sunlit greenhouse and watered regularly. After 28 days, all the plants were removed, washed in running tap water to remove soil particles and evaluated for disease incidence.

### Statistics and calculations of plant disease

The plant disease incidence under each treatment was calculated as the percentage of diseased plants. Disease severity was scored on a 0-4 scale (0 = peanut growth was normal, and the base of the stem had apparent disease spots; 1 = peanut growth was normal, and there were apparent disease spots at the base of the stem; 2 = less than 25% of the whole peanut plant had wilted or died; 3 = 26~50% of the whole peanut plant had wilted or died; 4 = greater than 50% of the whole peanut plant had wilted or died). The disease index and the relative biocontrol efficacy of the antagonists were calculated as follows [5, 14]:

$$\mathrm{Disease}\ \mathrm{in}\mathrm{d}\mathrm{ex}=100\times \sum \left(\mathrm{Number}\ \mathrm{of}\ \mathrm{disease}\mathrm{d}\ \mathrm{plants}\ \mathrm{in}\ \mathrm{each}\ \mathrm{grade}/\mathrm{Total}\ \mathrm{number}\ \mathrm{of}\ \mathrm{plants}\ \mathrm{in}\mathrm{vestigated}\ \mathrm{with}\ \mathrm{the}\ \mathrm{highest}\ \mathrm{disease}\ \mathrm{in}\mathrm{d}\mathrm{ex}\right)$$

$$\mathrm{Relative}\ \mathrm{biocontrol}\ \mathrm{efficacy}\left(\%\right)=\left(\mathrm{Disease}\ \mathrm{index}\ \mathrm{of}\ \mathrm{the}\ \mathrm{control}-\mathrm{Disease}\ \mathrm{index}\ \mathrm{of}\ \mathrm{the}\ \mathrm{antagonist}\right)/\mathrm{Disease}\ \mathrm{index}\ \mathrm{of}\ \mathrm{the}\ \mathrm{control}$$

The differences in data statistics and calculation were analysed by one-way analysis of variance (ANOVA) followed by Tukey’s pairwise post-hoc comparisons.

### Plot experiments

Plot experiments were employed to evaluate the biocontrol performance of *P. chlororaphis* zm-1 and its mutants against peanut stem rot. We carried out experiments according to a previously reported method [27] with some modifications. Specifically, the test plot was divided into 6 sections, each with a size of 4.0 × 3.2 m and surrounded by cement walls, after which the sifted soil was sterilized and added to the cement-divided plots. The soil consisted of Tifton loamy sand (fine-loamy, siliceous and thermic Plinthic Kandiudult; pH of 6.1) artificially infested with *S. rolfsii*. Peanut seeds were sown in each divided plot at a spacing of 40 × 20 cm. The main plots (12.8 × 7.6 m) consisted of four divided treatment plots and two untreated plots. Data were collected from the treated plots. The relative biocontrol efficacy was ultimately calculated as shown above.

## Results

### *P. chlororaphis* zm-1 inhibits the growth of *S. rolfsii* through extracellular compounds

**Fig. 1:**
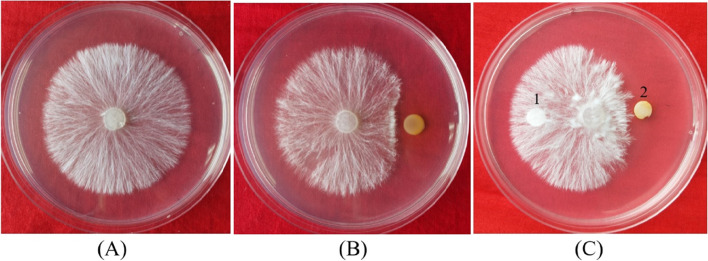
The results of our confrontation experiment. **A, B** Confrontation experiment between *P. chlororaphis* zm-1 and *S. rolfsii*; **C** The inhibited effects of PECE on the growth of *S. rolfsii*. 1 responds filter paper loaded with 20 μL methanol, and 2 responds that with 20 μL PECE

As shown in Fig. 1, *P. chlororaphis* zm-1 strongly inhibited *S. rolfsii* under our laboratory conditions (Fig. 1B). Due to the growth and antagonism of *P. chlororaphis* zm-1, the mycelium of *S. rolfsii* extended only 1.38 cm in the transverse direction. At the same time, they extended 3.78 cm along the vertical axis. In the control group without any interference from antagonistic strains, the mycelium of *S. rolfsii* extended 4.06 cm when they were cultured for the same time (Fig. 1A). PECE was prepared to investigate whether the extracellular products produced by *P. chlororaphis* zm-1 were the active substances inhibiting the growth of *S. rolfsii*. The inhibitory effect of PECE on *S. rolfsii* is shown in Fig. 1C. The aerial hyphae of *S. rolfsii* extended only 1.97 cm in the direction where a dish loaded with 30 μL of PECE was placed, but in the undisturbed direction, the hyphae stretched 3.24 cm. As shown in Fig. 1C, PECE significantly (*P* < 0.05) inhibited the mycelial expansion of *S. rolfsii*, while methanol, a negative control (NC), had no effect on the expansion of mycelia. These results indicated that *P. chlororaphis* zm-1 has great potential for use as a biocontrol agent to control peanut stem rot caused by *S. rolfsii*.

### Analysis of the antibacterial components of PECE via HPLC

**Fig. 2:**
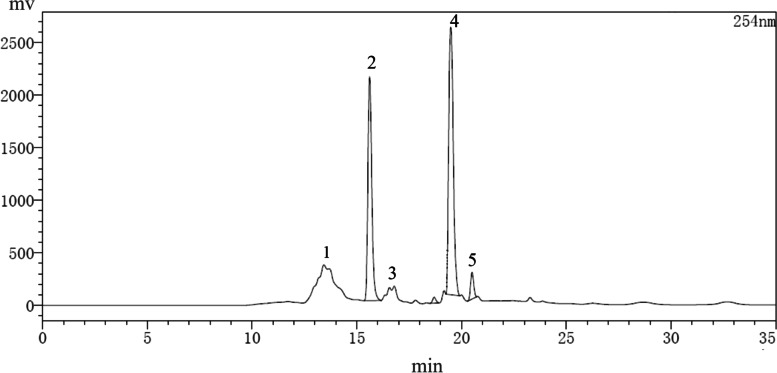
The liquid chromatogram of PECE. Number 1-5 respond the five components, respectively

**Fig. 3:**
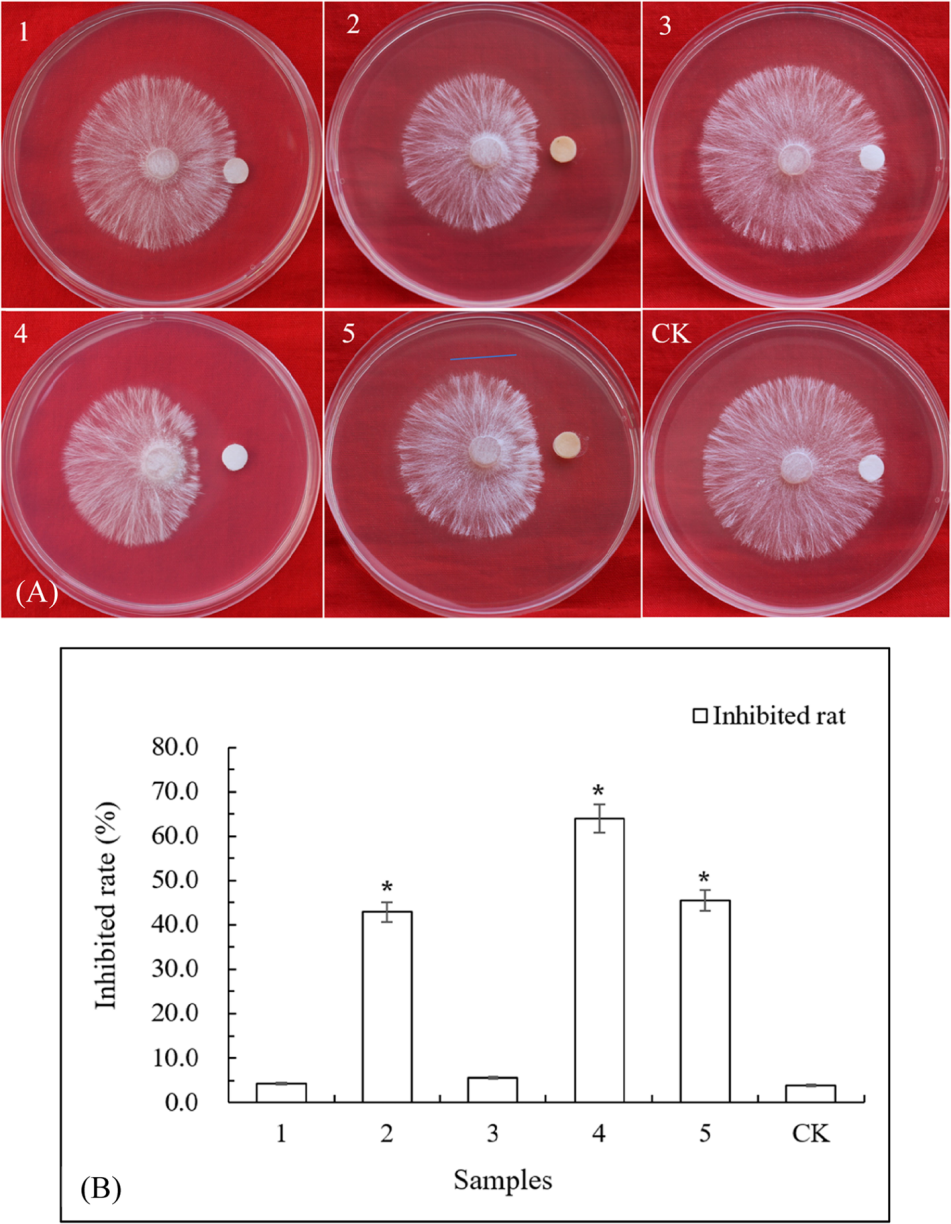
**A** The inhibited effects on the growth of *S. rolfsii* of each component in Fig. 2. Each filter paper loaded with 20 μL test sample, and methanol was used as blank control (CK). **B** The inhibited rates of each component in Fig. 2. *means the significant difference (*P* < 0.05)

The components of PECE as analysed by HPLC are shown in Fig. 2. There were 5 principal peaks in the liquid chromatogram. To confirm which component plays a key role in inhibiting the growth of *S. rolfsii*, we isolated the five components via HPLC and collected them separately (referred to as 1, 2, 3, 4, and 5) for identification of their antifungal activities. The results of our identification experiments were shown in Fig. 3. Components 2, 4 and 5 all inhibited the growth of *S. rolfsii*. We purchased three standard phenazines, 1-hydroxyphenazine, phenazine-1-carboxylic acid, and the core phenazine compound, each of their retention times was determined under the same chromatographic conditions. On the basis of the determination of retention time compared with the liquid chromatograms of the three standard reagents in Fig. S1, the components 2, 4 and 5 were very likely to be 1-hydroxyphenazine, phenazine-1-carboxylic acid, and the core phenazine compound, respectively.

### Identification of phenazines in PECE via UPLC-MS/MS

**Fig. 4:**
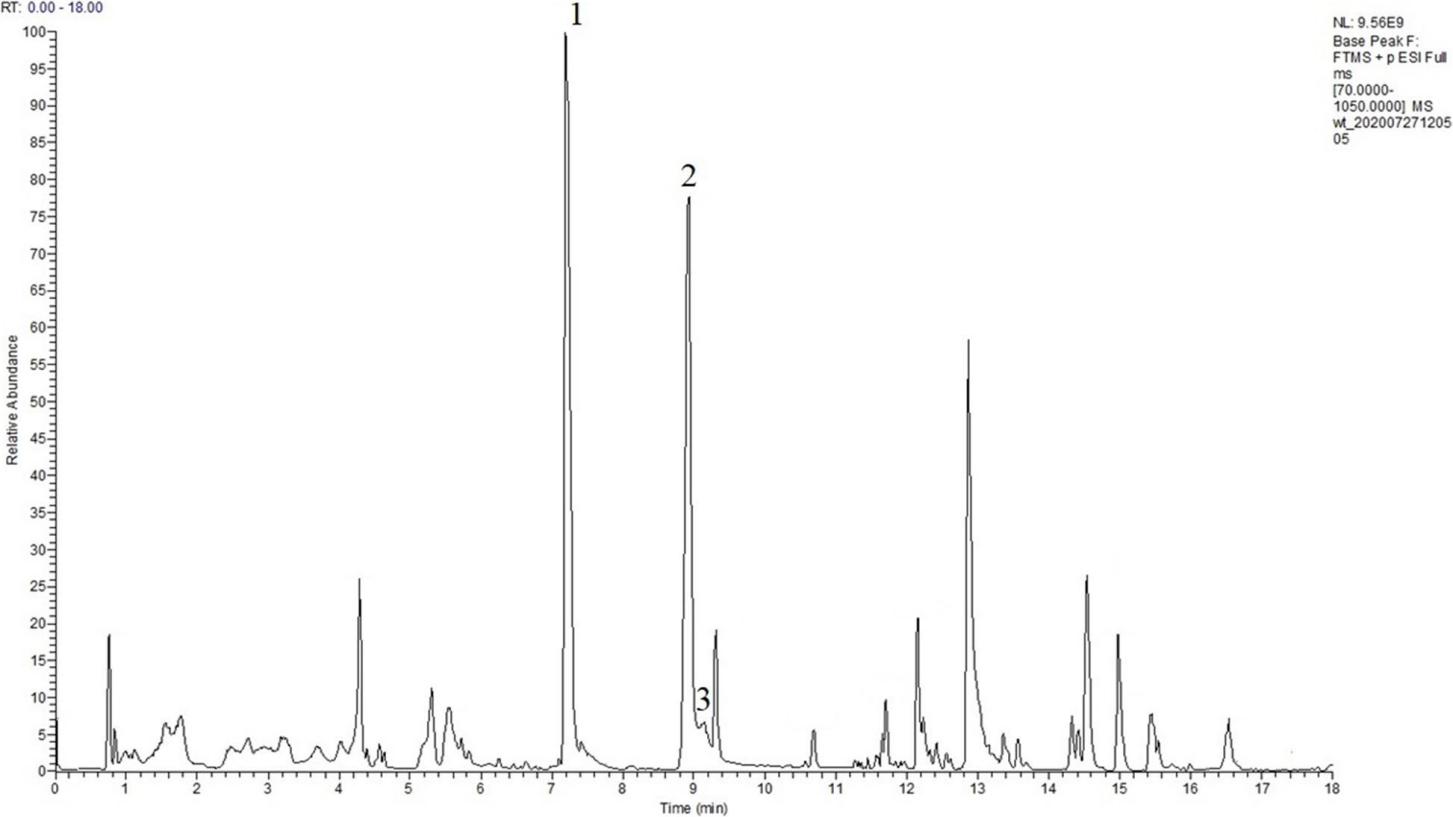
The total ion liquid chromatogram of the PECE. “1” responds 1- hydroxyphenanzine, “2” responds phenazine-1-carboxylic acid, “3” responds phenazine, all of them were identified by standard control

**Fig. 5:**
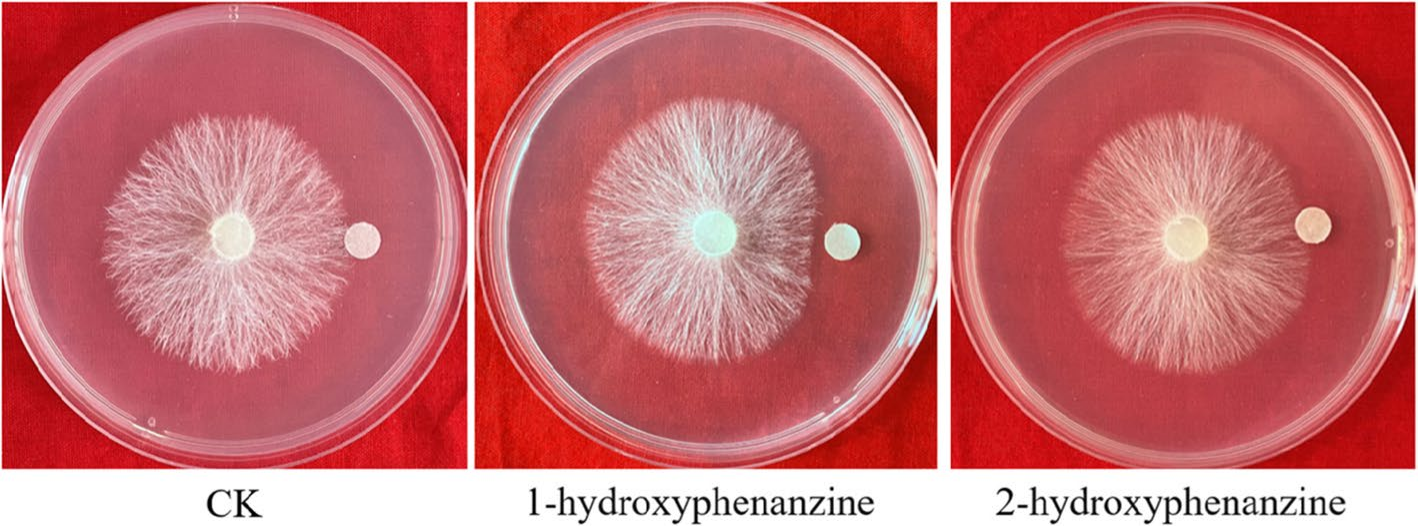
The inhibited effects on the growth of *S. rolfsii* of hydroxyphenanzine. Each filter paper loaded with 20 μL test sample, and methanol was used as blank control (CK). By calculating the inhibition rate of mycelial growth on each plate, only 1-hydroxyphenazine inhibited the mycelial growth of *S. Rolfsii* by 13.64%

To define component of PECE, UPLC-MS/MS was used to display the total ion liquid chromatogram. In Fig. 4, more than fifteen compounds were presented in the PECE. There are more than fifteen compounds present in the PECE. According to the above HPLC results, we focused on the determination of phenazine compounds in the PECE and identified three principal components, 1-hydroxyphenazine, phenazine-1-carboxylic acid, and the core phenazine, via alignment with database information. The details of the identification information were shown in Table 1, and the chromatograms obtained by mass spectrometry are shown in Supplementary Document 2. According to the UPLC-MS/MS results, peak 1 could be attributed to 1-hydroxyphenazine or 2-hydroxyphenazine. We further measured the antibacterial activity of standards 1-hydroxyphenazine and 2-hydroxyphenazine and found that 1-hydroxyphenazine could inhibit the growth of *S. rolfsii*, but 2-hydroxyphenazine could not (Fig. 5). Thus, combined with the experimental results in section 3.2, the peak 1 corresponded to 1-hydroxyphenazine. Therefore, *P. chlororaphis* zm-1 could produce three phenazines, 1-hydroxyphenazine, phenazine-1-carboxylic acid, and the core phenazine. We proposed that *P. chlororaphis* zm-1 antagonizes *S. rolfsii* by producing and secreting phenazine compounds.

**Table 1:** The details of phenazine compounds identified by UPLC-MS/MS

Peak No.	RT (min)	Molecular weight	Molecular formula	Δmass (ppm)	Tentative identification	mzVault Match
1	7.20	197.0705	C_12_H_8_N_2_O	-1.10	1- Hydroxyphenanzine	95.9
2	8.91	224.0587	C_17_H_32_O_3_	2.79	Phenazine-1-carboxylic acid	91.0
3	9. 11	181.0756	C_12_H_8_N_2_	-3.41	Phenazine	84

### Deletion of *the phzE* or *phzH* gene blocked phenazine compound production by *P. chlororaphis* zm-1

**Fig. 6:**
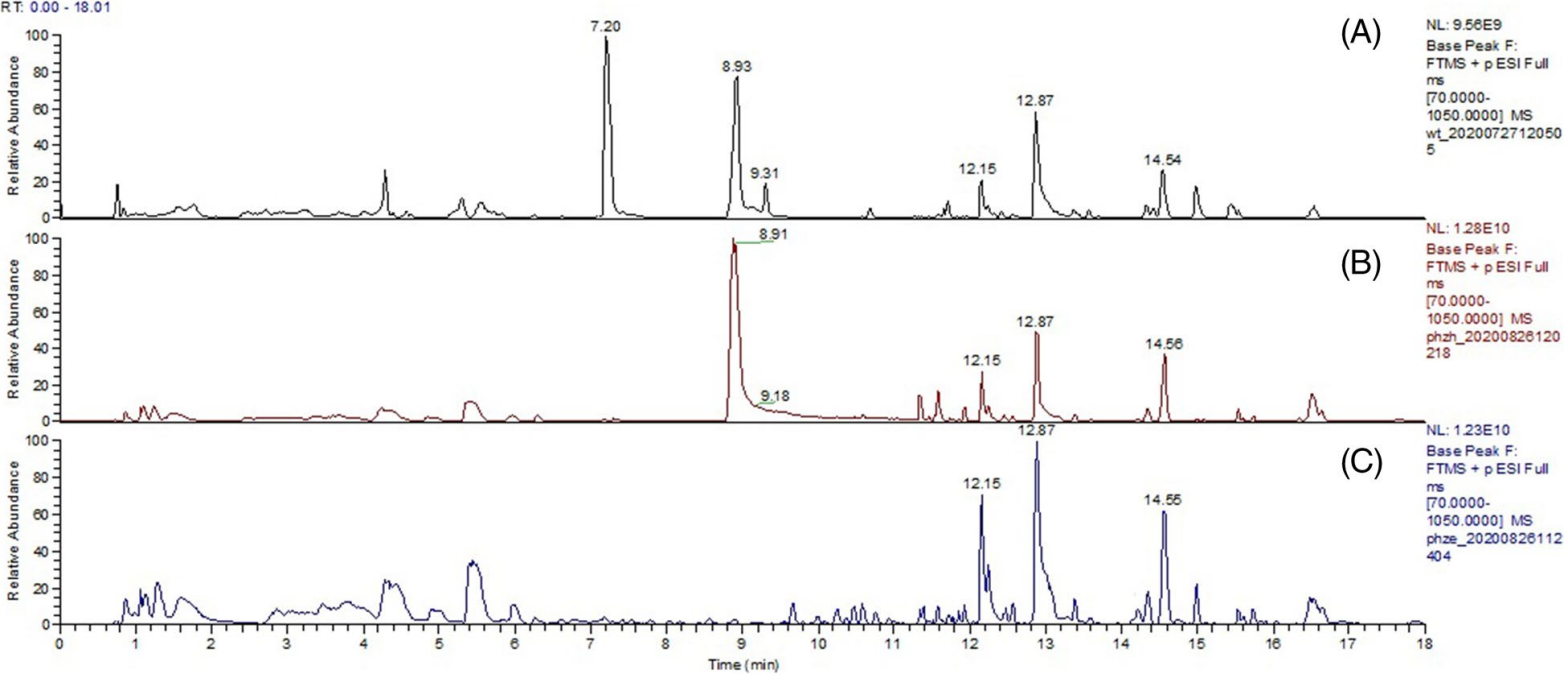
The total ion liquid chromatogram of the PECE from *P. chlororaphis* zm-1 and its mutants. **A** *P. chlororaphis* zm-1; **B** Δ*phzH*; **C** Δ*phzE*

According to the whole genome of *P. chlororaphis* zm-1 (GenBank ID: CP048051) and subsequent gene functional annotations, the *P. chlororaphis* zm-1 genome contain two gene products of phenazine biosynthesis protein (Protein ID: WP_038575848.1) and phenazine-specific anthranilate synthase (Protein ID: WP_081359794.1), respectively. Through blast on NCBI, we found the phenazine biosynthesis protein is highly homologous (100%) to an anthranilate synthase family protein from *P. chlororaphis*, named PhzH, which reported to be related to the phenazine-1-carboxylic acid-producing *Pseudomonas* spp. strains. And the phenazine-specific anthranilate synthase is highly homologous (100%) to phenazine biosynthesis protein from PhzE (100%), which were reported to be a highly conserved phenazine biosynthetic operon both in *P. chlororaphis* and *P. aeruginosa*. Therefore, we constructed the two gene mutants of *phzH* and *phzE*, and examined the biocontrol effects of *P. chlororaphis* zm-1 on abnormal synthesis of extracellular phenazine compound. Thus, we analysed the components of their extracellular compounds by UPLC-MS/MS. The total ion liquid chromatograms of the PECE from *P. chlororaphis* zm-1 and its mutants are shown in Fig. 6. As a result, *phzH* gene deletion inhibited the production of 1-hydroxyphenazine, but the synthesis of phenazine-1-carboxylic acid and phenazine was unaffected. More interestingly, Δ*phzE* could not produce any of the three kinds of phenazine compounds. This result indicated that both PhzE and PhzE – especially PhzE – were closely related to the synthesis of phenazine compounds in *P. chlororaphis* zm-1.

### Deletion of the *phzE* gene prevents *P. chlororaphis* zm-1 from antagonizing *S. rolfsii*

**Fig. 7:**
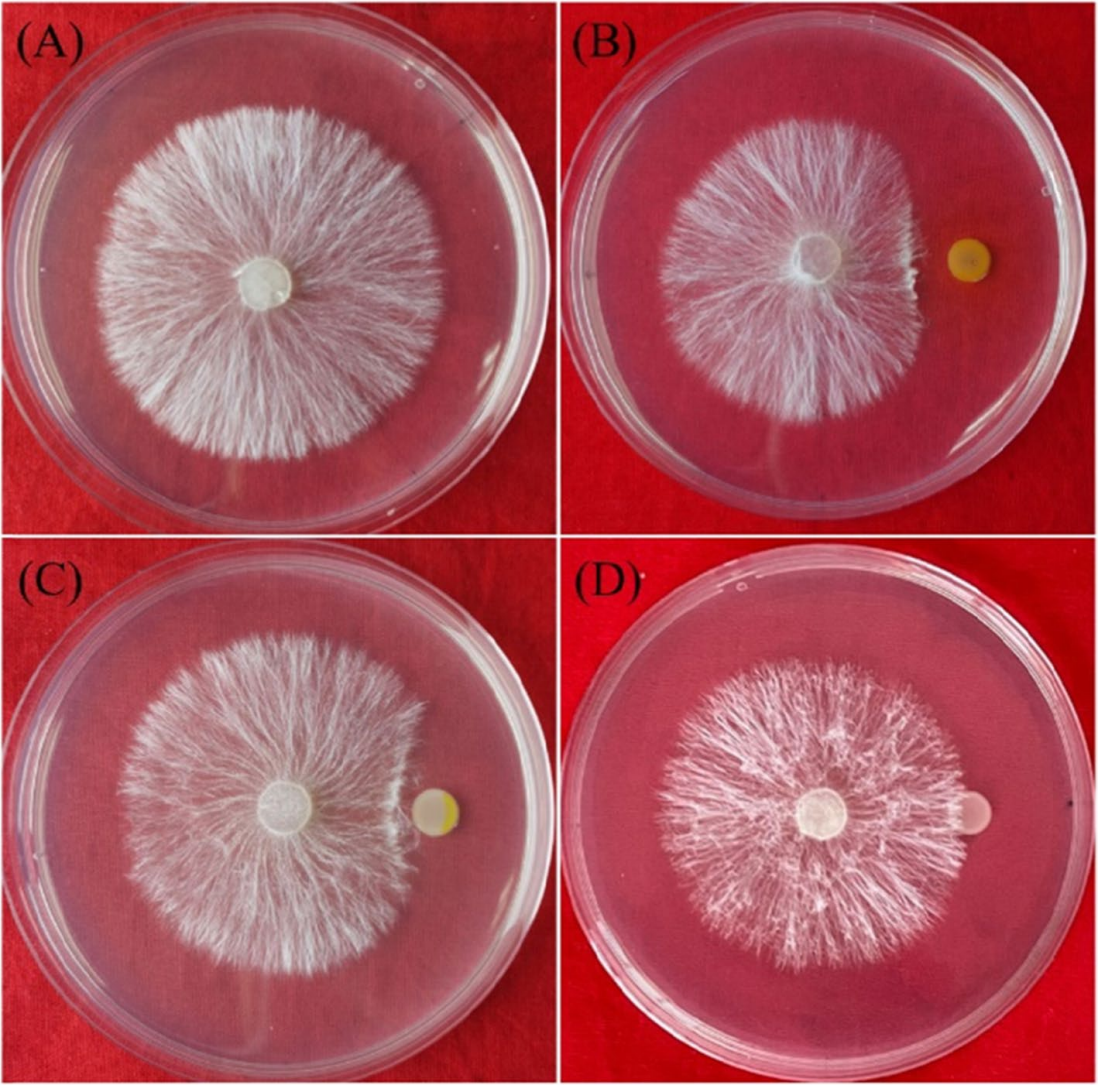
The antifungal activities of *P. chlororaphis* zm-1 and its mutants of Δ*phzH* and Δ*phzE*. **A** Control; **B** *P. chlororaphis* zm-1; **C** Δ*phzH* mutant; **D** Δ*phzE* mutant. By calculating the inhibition rate of mycelial growth on each plate, *P. chlororaphis* zm-1 inhibited the mycelial growth of *S. Rolfsii* by 42.86%. The inhibition rate of Δ*phzH* mutant decreased to 19.23%. But the Δ*phzH* mutant did not inhibit the growth of *S. Rolfsii*

To illustrate the mechanism through which *P. chlororaphis* zm-1 antagonizes *S. rolfsii*, we further determined the antifungal activities of Δ*phzH* and Δ*phzE*, and the results are shown in Fig. 7. Contrast to wild-type *P. chlororaphis* zm-1 (42.86%), the antifungal activities of the Δ*phzH* mutant decreased to 19.23%, and Δ*phzE* completely lost its antifungal activity. These results were in accordance with the results of the composition analysis above. Since Δ*phzE* could not produce any kind of phenazine compound (Fig. 6C), it lost antifungal activity against *S. rolfsii*. While Δ*phzE* lost the abilities to produce 2-OH- phenazine, but it still had PCA production, thus, its antifungal activity was just decreased. Therefore, we conclude that *P. chlororaphis* zm-1 antagonizes *S. rolfsii* by producing and secreting phenazine compounds under our experimental conditions.

### Biocontrol efficacy of *P. chlororaphis* zm-1 and its mutants in the greenhouse

The biocontrol efficacy of Δ*phzE* and Δ*phzH* in the greenhouse was determined, and the results were shown in Table 2. Compared to the sterile water control and fungicide control treatments, the *P. chlororaphis* zm-1 and Δ*phzH* treatments significantly reduced both the disease incidence and severity of peanut stem rot (*P* < 0.05). The biocontrol efficacy of *P. chlororaphis* zm-1 was significantly (*P* < 0.05) higher than that of carbendazim (70.60%), reaching 75.63%. However, the biocontrol efficacy of Δ*phzH* dramatically declined to 47.21%. The biocontrol efficacy of Δ*phzE* was reduced to 6.19%, and ΔphzE almost lost its ability to inhibit the growth of *S. rolfsii*. These results were in strongly in accordance with those of the above experiments, which confirmed that *P. chlororaphis* zm-1 antagonizes *S. rolfsii* by producing and secreting phenazine compounds.

**Table 2:** The biocontrol efficacy of *P. chlororaphis* zm-1 and its mutants in greenhouse

Treatment group	Incidence rate (%)	Disease index(%)	Biocontrol efficacy (%)
Negative Control (NC)	100 ± 0 ^a^	86.17 ± 0.5 ^a^	/
Carbendazim	54.0 ± 2.0 ^c^	25.3 ± 1.2 ^c^	70.60 ± 1.35 ^b^
*P. chlororaphis* ZM-1	44.0 ± 2.0 ^d^	21.0 ± 1.0 ^d^	75.63 ± 1.16 ^a^
Δ*phzH*	74.0 ± 2.0 ^b^	45.5 ± 1.5 ^b^	47.20 ± 1.71 ^c^
Δ*phzE*	99.0 ± 1.0 ^a^	80.8 ± 0.7 ^a^	6.19 ± 0.77 ^d^

Values are the mean ± SD. Different lowercase letters in the same column indicated a significant difference between the treatments (*P* < 0.05)

### Plots of the biocontrol efficacy of *P. chlororaphis* zm-1 and its mutants

Because the greenhouse assay results showed differences between *P. chlororaphis* zm-1, Δ*phzH* and Δ*phzE*, we further determined the biocontrol efficacy of Δ*phzH* and Δ*phzE* in actual plots, The results in Table 3 were in accordance with the trend of the greenhouse assay. The *P. chlororaphis* zm-1 and Δ*phzH* treatments significantly reduced both the disease incidence and the severity of peanut stem rot (*P* < 0.05), although the biocontrol efficacy of Δ*phzH* decreased, contrast to wild-type *P. chlororaphis* zm-1. The biocontrol efficacy of *P. chlororaphis* zm-1 was significantly (*P* < 0.05) higher than that of carbendazim (49.87%), reaching 57.09% in the plot experiments. Similarly, the biocontrol efficacy of Δ*phzE* was reduced to 1.88%; in fact it almost lost its biological control activity.

**Table 3:** The biocontrol efficacy of *P. chlororaphis* zm-1 and its mutants in plot

Treatment group	Incidence rate (%)	Disease index(%)	Biocontrol efficacy (%)
Negative Control (NC)	85.63 ± 0.63 ^a^	58.44 ± 0.96 ^a^	/
Carbendazim	47.82 ± 0.96 ^c^	29.3 ± 0.54 ^c^	49.87 ± 0.94 ^b^
*P. chlororaphis* ZM-1	40.00 ± 1.25 ^d^	25.08 ± 0.86 ^d^	57.09 ± 1.47 ^a^
Δ*phzH*	47.19 ± 0.94 ^b^	32.35 ± 0.93 ^b^	44.67 ± 1.62 ^c^
Δ*phzE*	84.38 ± 0.63 ^a^	57.35 ± 0.94 ^a^	1.88 ± 1.61 ^d^

Values are the mean ± SD. Different lowercase letters in the same column indicated a significant difference between the treatments (*P* < 0.05)

## Discussion

Peanut stem rot is a serious plant disease that causes great economic loss and has become increasingly severe in recent years. Biological control is one of the most promising plant disease control measures. In this study, we screened biocontrol *P. chlororaphis* strains from the rhizosphere soil of *A. asphodeloides* and identified *P. chlororaphis* zm-1 isolate against peanut stem rot. We sequenced its whole-genome sequence which was uploaded to the NCBI database (GenBank ID: CP048051.1). Under laboratory conditions, we confirmed that *P. chlororaphis* zm-1 had a strong antagonistic effect on *S. rolfsii* (Fig. 1A). The biological control of plant pathogens by *Pseudomonas* works through three main types of modes of action: direct interactions with the pathogen via antibiosis; interactions with the host plant via induction of resistance; and competition with the pathogen for nutrients and niches [21]. To investigate whether *P. chlororaphis* zm-1 antagonizes *S. rolfsii* by producing extracellular active substances, we extracted the supernatant of *P. chlororaphis* zm-1 fermentation broth with ethyl acetate and methanol, and identified that PECE significantly inhibited the growth of *S. rolfsii*, but methanol, a negative control (NC), had no effect on the expansion of mycelia. These results indicated that *P. chlororaphis* zm-1 has great potential for use as a biocontrol agent to control peanut stem rot caused by *S. rolfsii*.

Since the extracellular metabolites of *P. chlororaphis* zm-1 contain anti-micronuclear chemicals, we analysed the components of PECE by HPLC. To confirm which component plays a key role in inhibiting the growth of *S. rolfsii*, we isolated the five identified components via HPLC and collected them separately (referred to as 1, 2, 3, 4, and 5) for identification of their antifungal activities. Components 2, 4 and 5 all inhibited the growth of *S. rolfsii*. According to previous reports, phenazines, which compose a class of diffusible, heterocyclic compounds, are among the well-studied secondary metabolites shown to be important for biological control [16, 24, 31]. With standard phenazines, the three components, 1-hydroxyphenazine, phenazine-1-carboxylic acid (PCA), and the core phenazine compound were identified by UPLC-MS/MS. According to previous reports, natural phenazine compounds, which compose a class of diffusible, heterocyclic compounds, comprise a large group of pigmented, heterocyclic, and nitrogen-containing aromatic secondary metabolites produced and secreted almost exclusively by eubacteria [8, 16]. In another related development, more than 180 different naturally derived phenazines have been identified, and most of them are among the well-studied secondary metabolites shown to be important for biological control [9, 16, 20, 31]. *Pseudomonas* and *Streptomyces* have been reported to produce phenazine compounds [26, 31], but the role of components phenazine components 2-hydroxyphenazine, PCA, and 1-OH-PCA was unclear. Thus, we speculate that the mechanism through which *P. chlororaphis* zm-1 antagonizes *S. rolfsii* may be related to its production of extracellular phenazine compounds. In a previous report by Le et al. [13], phenazine-producing *P. chlororaphis* strain Phz24 significantly inhibited the hyphal growth of *S. rolfsii* and suppressed stem rot disease in peanut, which was related to its phenazine components 2-hydroxyphenazine, PCA, and 1-OH-PCA, but which component played the major role is unclear. Our results further confirmed the antifungal activities of 1-hydroxyphenazine, PCA, and phenazine against *S. rolfsii*. In particular, 1-OH-PHZ produced by *P. chlororaphis* zm-1 also showed antifungal activities against *S. rolfsii*, but the previously reported 2-OH-PHZ did not (Fig. 5).

The metabolic regulatory pathways of phenazines have been extensively studied during the past several years. Through comparisons of the *phz* operons in different species, five conserved sets of enzymes, PhzE, PhzD, PhzF, PhzB, and PhzG, have been confirmed to be present in all phenazine-producing bacteria [8, 20, 23]. By reports, in most phenazine produced *pseudomonas*, the phenazine biosynthetic operon is flanked by one or more coding genes whose coding products are responsible for converting PCA to different phenazine derivatives by methylation, transamination, hydroxylation, or decarboxylation [6, 16]. According to the report of Müller and Behrendt [21], PhzE plays a key role in the synthesis of phenazine compounds from *Pseudomonas* spp. In another two reports of Mavrodi et al. [16, 17], the genome of the opportunistic human pathogen *P. aeruginosa* PAO1 contained several phenazine-modifying genes whose products could convert PCA into three additional phenazine derivatives, i.e., PCN, 5-methyl-phenazine-1-carboxylic acid, and 1-hydroxy-phenazine, via the activity of *phzH*, *phzM*, and *phzS*, respectively. PhzH is likely to participate in converting PCA into additional phenazine derivatives [16, 17].

We sequenced the whole genome of *P. chlororaphis* zm-1, the sequencing results (GenBank ID: CP048051) and subsequent gene functional annotations showed that the *P. chlororaphis* zm-1 genome contain two gene products which are annotated as a phenazine biosynthesis protein (Protein ID: WP_038575848.1) and phenazine-specific anthranilate synthase (Protein ID: WP_081359794.1), respectively. We downloaded their amino acid sequence and blast on NCBI, then, we found that the phenazine biosynthesis protein is highly homologous (100%) to an anthranilate synthase family protein from *P. chlororaphis*, named PhzH, which could extend the range of biocontrol ability of phenazine-1-carboxylic acid-producing *Pseudomonas* spp. strains by the previous report of Chin-A-Woeng et al. [3]. And the phenazine-specific anthranilate synthase is highly homologous (100%) to phenazine biosynthesis protein, PhzE, which were reported to be a highly conserved phenazine biosynthetic operon both in *P. chlororaphis* and *P. aeruginosa* [31]. Therefore, we constructed the two gene mutants of Δ*phzH* and Δ*phzE*, and examined the biocontrol effects of *P. chlororaphis* zm-1 on abnormal synthesis of extracellular phenazine compounds. First, we constructed two gene mutants, Δ*phzH* and Δ*phzE*, and determined their antifungal activities (Fig. 7). In contrast to those of wild-type *P. chlororaphis* zm-1, the antifungal activities of the Δ*phzH* mutant decreased, and Δ*phzE* lost its antifungal activity. We further analysed the composition of their extracellular compounds by UPLC-MS/MS and found that Δ*phzE* could not produce all three kinds of phenazine compounds and that deletion of the *phzH* gene inhibited the production of 1-hydroxyphenazine; however, the synthesis of phenazine-1-carboxylic acid and phenazine was unaffected. A previous report indicated that *phzH*, a phenazine biosynthesis-related gene, was identified in *P. chlororaphis* PCL1391 and shown to be required for the presence of the 1-carboxamide group of PCN because a *phzH* mutant of strain PCL1391 accumulated PCA [3]. However, our results showed that the Δ*phzH* mutant of *P. chlororaphis* zm-1 also accumulated PCA, but PhzH was required for the production of 1-hydroxyphenazine, which is not quite consistent with the findings concerning *P. chlororaphis* PCL1391. This is very likely due to the differences in extracellular metabolites of different strains under different culture conditions. Therefore, we conclude that *P. chlororaphis* zm-1 antagonizes *S. rolfsii* by producing and secreting phenazine compounds under our experimental conditions.

To confirm the mechanism through which *P. chlororaphis* zm-1 antagonizes *S. rolfsii*, we further determined the biocontrol efficacy of Δ*phzH* and Δ*phzE* via greenhouse and plot experiments. As a result, *P. chlororaphis* zm-1 significantly reduced both the disease incidence and the severity of peanut stem rot (*P* < 0.05) in both greenhouse assay and plot experiments. The biocontrol efficacy of *P. chlororaphis* zm-1 was significantly (*P* < 0.05) higher than that of carbendazim. Moreover, Δ*phzH* treatments also significantly reduced both the disease incidence and the severity of peanut stem rot (*P* < 0.05), but the biocontrol efficacy of Δ*phzH* dramatically declined, contrast to the wild-type strain. And Δ*phzE* mutant almost completely lost the abilities to inhibit *S. rolfsii*. These results were strongly in accordance with those of the above experiments, which confirmed that *P. chlororaphis* zm-1 antagonizes *S. rolfsii* by producing and secreting phenazine compounds.

The biocontrol mechanism of *P. chlororaphis* zm-1 is an important theoretical basis for its application in the control of peanut stem rot disease. Biocontrol mechanisms are one of the main ways for biocontrol strains to exert their control. In addition, it has been reported that *P. chlororaphis* can induce hosts to form a self-defence mechanism against pathogens. Whether the biocontrol effect of *P. chlororaphis* zm-1 on peanut stem rot is also related to this mechanism is still unclear. This will be our main topic in investigate in terms of the degree of the biocontrol of this strain in future experiments.

## Conclusion

In this study, we found *P. chlororaphis* zm-1 can antagonize *S. rolfsii*, which is the main causal agent of peanut stem rot through producing extracellular antifungal substances, Thus, *P. chlororaphis* zm-1 could prevent and control peanut stem rot disease. Furthermore, we identified the extracellular antifungal substances were 1-hydroxyphenazine, phenazine-1-carboxylic acid, and the core phenazine compound. Moreover, 1-hydroxyphenazine inhibited the growth of *S. rolfsii*, but 2-hydroxyphenazine did not. Δ*phzE* did not produce any phenazine compounds, and its antagonistic ability was almost completely abolished. Relatedly, Δ*phzH* no longer produced 1-hydroxyphenazine but could still produce PCA and phenazine, and its antagonistic ability declined. Therefore, our results showed *phzE* in *P. chlororaphis* zm-1 was essential for production of the active phenazine compounds, while *phzH* was mainly associated with PCA and phenazine productions. With potting experiments under greenhouse conditions and plot experiments, *P. chlororaphis* zm-1 all showed the biocontrol ability in controlling peanut stem rot antagonize *S. rolfsii*. Our findings provide a new idea for the effective prevention and treatment of peanut stem rot.

## Supplementary Information

**Additional file 1: Table S1.** The primers used in this text. **Table S2.** The number of diseased plants in each grade. **Table S3.** The biocontrol efficacy of each group tested strains in greenhouse experiments. **Table S4.** The number of diseased plants in each grade. **Table S5.** The biocontrol efficacy of each group tested strains in plot experiments. **Figure S1.** The liquid chromatograms of the three standard reagents.

**Additional file 2: Figure 1.** The total ion flow chromatogram of the PECE. **Figure 2.** The mass spectra corresponding to chromatographic peaks with retention time of 7.20. **Figure 3.** The mass spectra corresponding to chromatographic peaks with retention time of 8.91. **Figure 4.** The mass spectra corresponding to chromatographic peaks with retention time of 9.11.

## Data Availability

Data on the genomes of *P. chlororaphis* subsp. *aurantiaca* zm-1 have been submitted to the Gene Bank of NCBI which is open, and its GenBank ID is CP048051.1. The website is as follow: https://www.ncbi.nlm.nih.gov/nucleotide/CP048051.1. And all the other data generated during this study are included in this published article and its supplementary information files.

## Abbreviations

PECE: Extracellular antibacterial compound
PCA: Phenazine-1-carboxylic acid
PDA: Potato dextrose agar
PCN: Phenazine-1-carboxamide
