# Supplementary material for: Characteristics of biological control and mechanisms of Pseudomonas chlororaphis zm-1 against peanut stem rot
Source: BMC Microbiol. 2022 Jan 5;22:9. doi: 10.1186/s12866-021-02420-x (PMC8729073; doi:10.1186/s12866-021-02420-x)
Supplement: Supplementary file 1 — Additional file 1: Table S1. The primers used in this text. Table S2. The number of diseased plants in each grade. Table S3. The biocontrol efficacy of each group tested strains in greenhouse experiments. Table S4. The number of diseased plants in each grade. Table S5. The biocontrol efficacy of each group tested strains in plot experiments. Figure S1. The liquid chromatograms of the three standard reagents. [file 12866_2021_2420_MOESM1_ESM.docx]

Table S1 The primers used in this text.

| Primer name | Sequence (5' to 3') | Base number |
| --- | --- | --- |
| *phzE*-up-*EcoR*I-s | ACACGAATTCACCTGGAAACCACCCCTGACGA | 32 |
| *phzE*-up-*BamH*I-a | ACACGGATCCGAGCCGGTTGCAGGATGCGCTC | 32 |
| *phzE*-down-*BamH*I-s | ACACGGATCCCTGATCCACACACCTGTCGAGA | 32 |
| *phzE*-down-*Hind*III-a | CACAAAGCTTCATAGAACGATGGTCCCCCGTC | 32 |
| *phzH*-up-*EcoR*I-s | ACACGAATTCCCGATCCCATGAGCGTGCTGCA | 32 |
| *phzH*-up-*BamH*I-a | CACAGGATCCCTGCACTTTTCAGATATTTACG | 32 |
| *phzH*-down-*BamH*I-s | ACACGGATCCAAGCCTGATACGCCGCATGGTG | 32 |
| *phzH*-down-*Hind*III-a | CACAAAGCTTGCGGTTTCGCGACCGTCATAAG | 32 |

| Treatment group | Grade 1 | Grade 2 | Grade 3 | Grade 4 |
| --- | --- | --- | --- | --- |
| Negative Control | 0 | 6 | 16 | 28 |
|  | 0 | 7 | 14 | 29 |
|  | 0 | 4 | 19 | 27 |
| Carbendazim | 14 | 5 | 3 | 4 |
|  | 16 | 4 | 6 | 2 |
|  | 15 | 3 | 4 | 5 |
| *P. chlororaphis* ZM-1 | 12 | 2 | 5 | 3 |
|  | 11 | 2 | 4 | 4 |
|  | 14 | 3 | 4 | 2 |
| Δ*phzH* | 10 | 7 | 11 | 9 |
|  | 13 | 6 | 9 | 10 |
|  | 12 | 5 | 10 | 9 |
| Δ*phzE* | 2 | 8 | 17 | 23 |
|  | 4 | 7 | 13 | 26 |
|  | 3 | 6 | 16 | 25 |

Table S2 The number of diseased plants in each grade.

Table S3 The biocontrol efficacy of each group tested strains in greenhouse experiments.

| Treatment group | Incidence rate (%) | Disease index（%） | Relative biocontrol efficacy (%) |
| --- | --- | --- | --- |
| Negative Control (NC) | 100 | 86.0 | / |
|  | 100 | 86.5 |  |
|  | 100 | 86.0 |  |
| Carbendazim | 52.0 | 24.5 | 71.57 |
|  | 56.0 | 25.0 | 70.99 |
|  | 54.0 | 26.5 | 69.25 |
| *P. chlororaphis* ZM-1 | 44.0 | 21.5 | 75.05 |
|  | 42.0 | 21.5 | 75.05 |
|  | 46.0 | 20.0 | 76.79 |
| Δ*phzH* | 74.0 | 46.5 | 46.04 |
|  | 76.0 | 46.0 | 46.62 |
|  | 72.0 | 44.0 | 48.94 |
| Δ*phzE* | 100 | 80.5 | 6.58 |
|  | 98.0 | 80.5 | 6.58 |
|  | 98.0 | 81.5 | 5.42 |

| Treatment group | Grade 1 | Grade 2 | Grade 3 | Grade 4 |
| --- | --- | --- | --- | --- |
| Negative Control | 21 | 34 | 45 | 36 |
|  | 18 | 40 | 38 | 42 |
| Carbendazim | 14 | 28 | 23 | 13 |
|  | 16 | 20 | 28 | 11 |
| *P. chlororaphis* ZM-1 | 12 | 23 | 16 | 15 |
|  | 11 | 18 | 24 | 9 |
| Δ*phzH* | 10 | 17 | 31 | 19 |
|  | 13 | 16 | 24 | 21 |
| Δ*phzE* | 22 | 31 | 47 | 34 |
|  | 18 | 37 | 43 | 38 |

Table S4 The number of diseased plants in each grade.

Note: The total number of each group：160*2=320

| Treatment group | Incidence rate (%) | Disease index（%） | Relative biocontrol efficacy (%) |
| --- | --- | --- | --- |
| Negative Control (NC) | 86.25 | 57.5 | / |
|  | 85.0 | 59.38 |  |
| Carbendazim | 48.75 | 29.84 | 48.93 |
|  | 46.88 | 28.75 | 50.80 |
| *P. chlororaphis* ZM-1 | 41.25 | 25.94 | 55.62 |
|  | 38.75 | 24.22 | 58.56 |
| Δ*phzH* | 48.13 | 33.28 | 43.05 |
|  | 46.25 | 31.41 | 46.26 |
| Δ*phzE* | 83.75 | 56.41 | 3.48 |
|  | 85.00 | 58.28 | 0.27 |

Table S5 The biocontrol efficacy of each group tested strains in plot experiments.


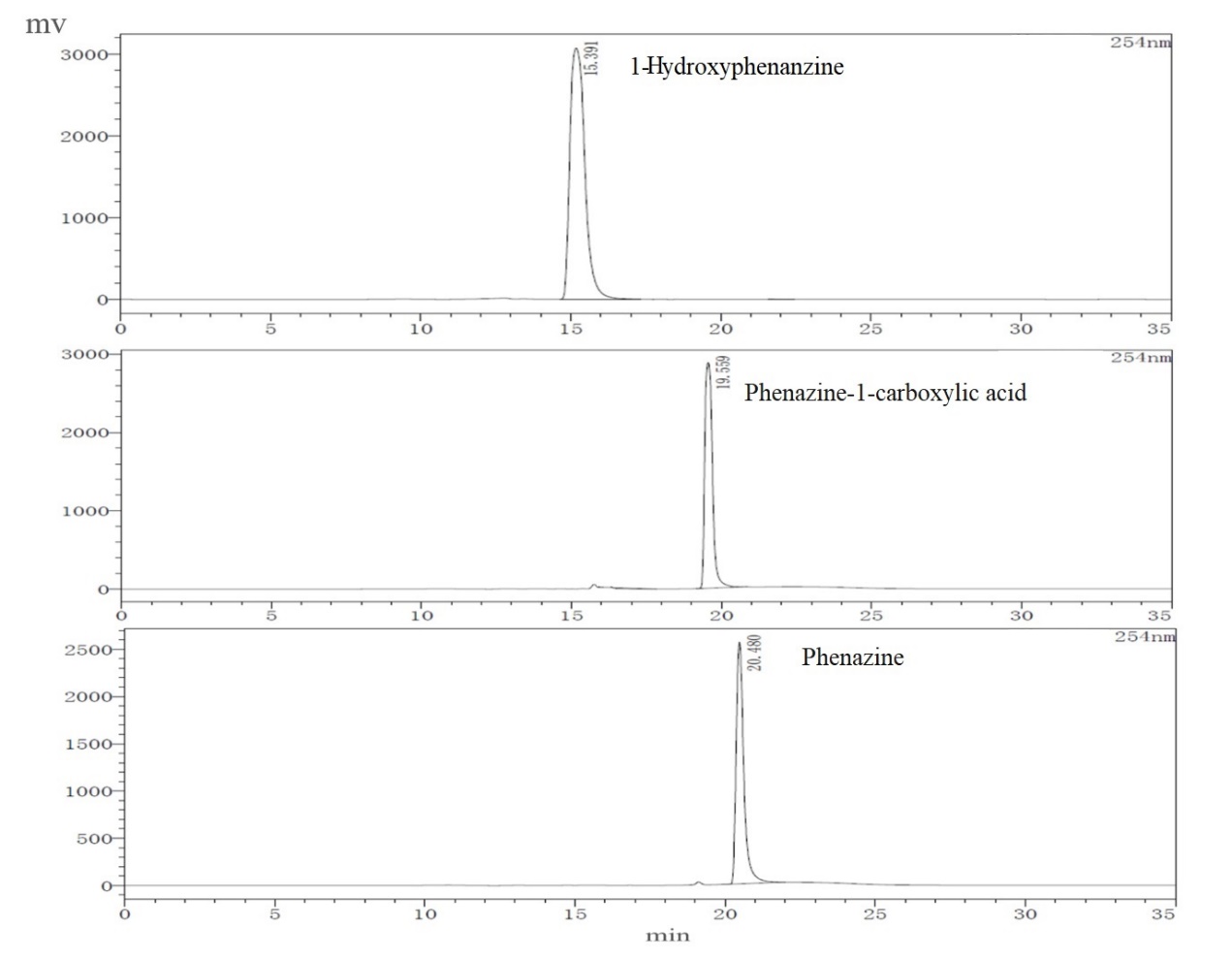


Figure S1 The liquid chromatograms of the three standard reagents.
