# Supplementary material for: Characteristics of biological control and mechanisms of Pseudomonas chlororaphis zm-1 against peanut stem rot
Source: BMC Microbiol. 2022 Jan 5;22:9. doi: 10.1186/s12866-021-02420-x (PMC8729073; doi:10.1186/s12866-021-02420-x)
Supplement: Supplementary file 2 — Additional file 2: Figure 1. The total ion flow chromatogram of the PECE. Figure 2. The mass spectra corresponding to chromatographic peaks with retention time of 7.20. Figure 3. The mass spectra corresponding to chromatographic peaks with retention time of 8.91. Figure 4. The mass spectra corresponding to chromatographic peaks with retention time of 9.11. [file 12866_2021_2420_MOESM2_ESM.docx]

Figure 1 The total ion flow chromatogram of the PECE.

Figure 2 The mass spectra corresponding to chromatographic peaks with retention time of 7.20

Figure 3 The mass spectra corresponding to chromatographic peaks with retention time of 8.91.


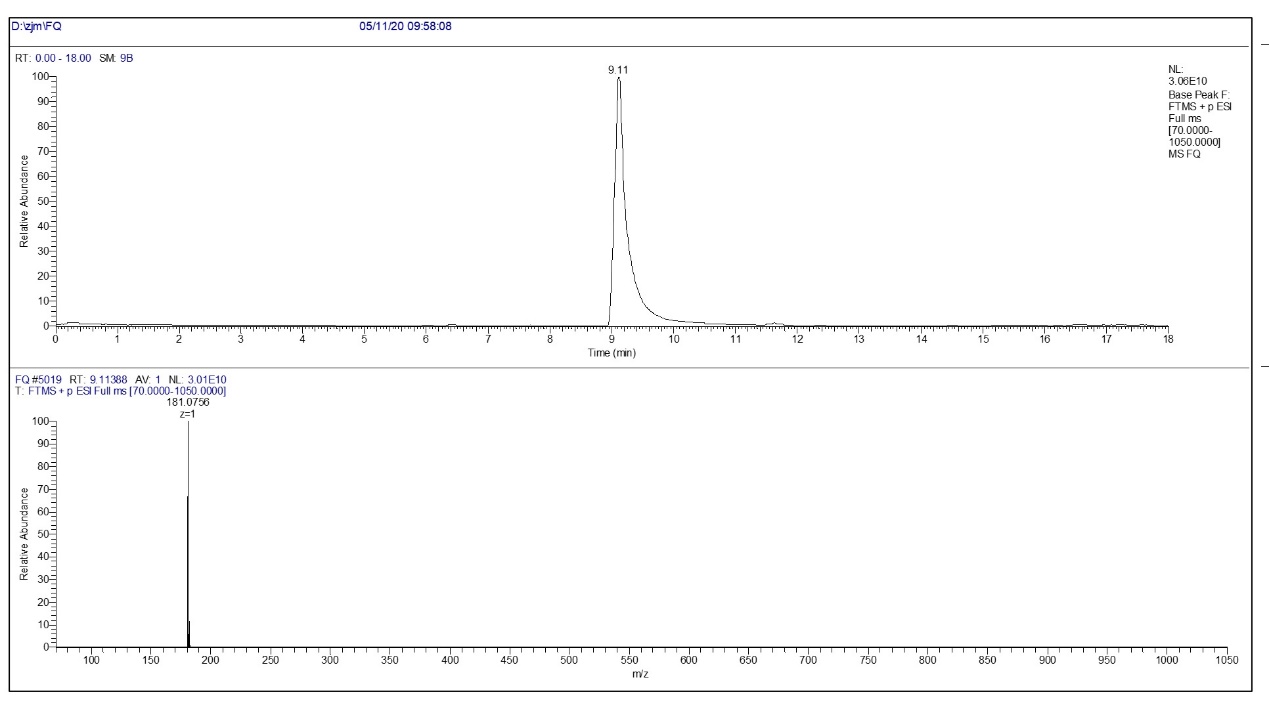


Figure 4 The mass spectra corresponding to chromatographic peaks with retention time of 9.11.
